# Prevalence of Peri-Implant Mucositis and Peri-Implantitis in Patients Treated with a Combination of Axial and Tilted Implants Supporting a Complete Fixed Denture

**DOI:** 10.1155/2015/874842

**Published:** 2015-05-06

**Authors:** Nicolò Cavalli, Stefano Corbella, Silvio Taschieri, Luca Francetti

**Affiliations:** ^1^Department of Biomedical, Surgical and Dental Sciences, IRCCS Istituto Ortopedico Galeazzi, Università Degli Studi di Milano, Via R. Galeazzi 4, 20161 Milan, Italy; ^2^Department of Biomedical, Surgical and Dental Sciences, Research Center in Oral Implantology, IRCCS Istituto Ortopedico Galeazzi, Università Degli Studi di Milano, Via R. Galeazzi 4, 20161 Milan, Italy; ^3^Department of Biomedical, Surgical and Dental Sciences, Research Center for Oral Health, IRCCS Istituto Ortopedico Galeazzi, Università Degli Studi di Milano, Via R. Galeazzi 4, 20161 Milan, Italy

## Abstract

*Objectives*. The aim of this retrospective study was to assess the incidence and prevalence of peri-implant mucositis and peri-implantitis in patients with a fixed full-arch prosthesis supported by two axial and two tilted implants.* Materials and Methods*. Sixty-nine patients were included in the study. Each patient received a fixed full-arch prosthesis supported by two mesial axial and two distal tilted implants to rehabilitate the upper arch, the lower arch, or both. Three hundred thirty-six implants for 84 restorations were delivered. Patients were scheduled for follow-up visits every 6 months in the first 2 years and yearly after. At each follow-up visit peri-implant mucositis and peri-implantitis were diagnosed if present.* Results*. The overall follow-up range was from 12 to 130 months (mean 63,2 months). Three patients presented peri-implantitis. The prevalence of peri-implant mucositis ranged between 0 and 7,14% of patients (5,06% of implants) while the prevalence of peri-implantitis varied from 0 to 4,55% of patients (3,81% of implants).* Conclusions*. The prevalence and incidence of peri-implant mucositis and peri-implantitis are lower than most of the studies in literature. Therefore this kind of rehabilitation could be considered a feasible option, on the condition of adopting a systematic hygienic protocol.

## 1. Introduction

Implant therapy is a consolidated procedure for full and partial rehabilitation of edentulous arches and this was widely supported by a large number of prospective studies with long-term follow-up [1–3].

However, the availability of bone volume could be an important factor that influences the possibility of achieving an adequate restoration through implant placement. In fact, in cases of severe bone atrophy, the available bone may not be sufficient for implant placement, requiring the adoption of bone grafting procedures [4]. Even though bone grafting procedures could be associated with high success rates, as reported by a number of studies, many complications and adverse sequelae could occur due to the demanding surgical procedure [5].

About 10 years ago Maló and coworkers [6, 7] described a treatment procedure that consists in an immediately loaded full-arch fixed prostheses supported by two mesial axial and two distal tilted implants, avoiding the adoption of bone graft procedures in lateroposterior area of mandibular and maxillary bone. This procedure was validated by scientific literature in terms of implant survival and success both in short and medium term, with a comparable bone resorption between axial and tilted implants [8, 9].

Despite high survival rates of restorations, the abovementioned surgical technique showed the susceptibility to biological and technical complications such as veneer fractures, soft tissue complications, abutment or screw loosening, loss of access hole restoration, and loss of retentions which were frequent [10, 11].

Late biological complications following dental implant therapy, consisting in peri-implant mucositis and peri-implantitis, nowadays are receiving increasing interest in the scientific literature.

Peri-implant mucositis can be described as a reversible inflammatory reaction of the soft tissues surrounding an implant whereas peri-implantitis can be identified by inflammatory reactions associated with bone loss around the implant [12].

The 6th and 7th workshops of periodontology suggested the clinical definition of peri-implant mucositis as the presence of bleeding on probing without loss of supporting bone. Peri-implantitis was defined as bleeding on probing, probing depth > 4 mm, and peri-implant bone loss [13, 14].

Heitz-Mayfield and Mombelli in a systematic review in 2014, although the currently available evidence does not allow any firm specific recommendation for the treatment of peri-implantitis, stated that some elements of therapy seem to be beneficial [15]. Those elements are oral hygiene instruction and counselling for smoking cessation, assessment of the prosthesis for access for plaque control, prosthesis removal and adjustments if required, nonsurgical debridement, surgical access to allow cleaning of the contaminated implant, and stabilization of the intraosseous peri-implant defect with a bone substitute/bone graft/bioactive substance with or without barrier membrane [15].

Recent reviews show that there is still lack of literature about the epidemiology of peri-implant mucositis and peri-implantitis in different types of rehabilitations, and the high quality studies presented contrasting results [16–20].

The aim of this retrospective study was to assess the incidence and prevalence of peri-implant mucositis and peri-implantitis in immediate full arch rehabilitations supported by two axial and two tilted implants.

## 2. Materials and Methods

This retrospective clinical study was conducted according to the principles embodied in the Helsinki Declaration of 1975 for biomedical research involving human subjects, as revised in 2000 [21]. The research project was approved by the review board of the IRCCS Istituto Ortopedico Galeazzi (RC 4.73).

### 2.1. Clinical Chart Selection

Clinical chart of treated patients were selected on the basis of the following criteria.

- The first criterion is patients treated with an immediate loading restoration following the All-on-Four treatment protocol as described first by Maló [6, 7] and slightly modified by Francetti et al. [22]. Briefly, firstly conservative and periodontal treatments were performed. Then all hopeless teeth, if present, were extracted and a regularization of the edentulous bone ridge was completed. Four implants (Branemark System MKIV or Nobel-Speedy Groovy, Nobel Biocare, Zurich, Switzerland) were positioned with the two anterior axial implants and the distal implants tilted by approximately 30 degrees with respect to the occlusal plane. To allow an immediate rehabilitation, each implant was inserted with a final torque of 40 to 50 Ncm. Straight and angulated Multi-Unit Abutments (MUA, Nobel Biocare AB) were connected to the implants. An impression was taken using a silicon putty polyvinylsiloxane directly on the coping and within 48 hours from surgery a temporary prosthesis was delivered. After 3 months of loading for the lower arch and 6 months of loading for the upper arch, in the absence of pain and inflammatory signs, the patients received the definitive prosthesis.
- The second criterion is maxillary or mandibular restoration.
- The third criterion is presence of clinical information about bleeding index (BI), plaque index (PI) [23], and probing depth at implant level (PD), retrieved in each follow-up visit.
- The fourth criterion is presence of periapical radiographs investigating bone resorption rate.

Clinical charts of patients that did not attend even one follow-up visit were excluded from the study.

### 2.2. Outcomes

All patients were scheduled for follow-up visits every 6 months in the first 2 years after surgery and yearly after.

Each patient received professional oral hygiene treatment and detailed oral hygiene instructions following the protocol proposed by Corbella et al. [23].

Once medical records included were retrieved, the following parameters were considered: bleeding index [23], plaque index [23], and probing depth. Probing depth was measured using a plastic probe (Color-vue Hu-Friedy, Rotterdam, Belgium, with University of North Carolina markings) with a probing force of 0.25 N [23]. The prosthesis was removed during each follow-up in order to access the area to probe. All radiographs were evaluated.

Incidence and prevalence of biological complications were the primary outcomes.

An implant was considered affected by peri-implant mucositis if it had at least one site with bleeding index > 1, and it was considered affected by peri-implantitis if it had at least one site with bleeding index > 1, probing depth > 4 mm, and radiographically detectable bone loss.

The related standardized medical records, including radiographic evaluation of all the surgical and prosthetic procedures, dental Ct scans, and clinical description that were included in each case file, were obtained, reviewed, and analysed by 3 authors independently (NC, SC, and ST). Cases of disagreement were jointly discussed until an agreement was achieved.

### 2.3. Statistical Analysis

Data about prevalence and incidence of peri-implant mucositis and peri-implantitis were presented through descriptive statistics. Percentage of implants and patients affected at a certain follow-up were calculated.

## 3. Results

An amount of 69 patients (29 male and 40 female patients; mean age 59,7 years; range from 40 to 84 years) was recruited. Forty patients received a mandibular restoration, 14 received a maxillary restoration, and 15 received both.

A total amount of 336 implants was placed (168 axial and 168 tilted) and 84 full arch restorations (55 mandibular and 29 maxillary) were delivered.

Eight mandibular prostheses were supported by Branemark System MKIV implants (Nobel Biocare, Zurich, Switzerland) (32 implants) while the other 47 mandibular and 29 maxillary prostheses were supported by Nobel-Speedy Groovy implants (Nobel Biocare, Zurich, Switzerland) (304 implants).

Ten patients did not come to all the follow-up visits expected by this study protocol and were excluded from the study. They were considered until the last follow-up visit before being dropped out.

Forty-nine restorations were delivered to nonsmoking patients, 14 to light-smokers (less than 12 cigarettes/day), and 21 to heavy-smokers (more than 12 cigarettes/day).

The 79,8% of the considered rehabilitations were positioned in patients with history of periodontitis and the 20,2% in patients with no history of periodontitis.

The opposing arch of mandibular restorations was a complete removable denture in the 34,6% of cases, an All-on-four prosthesis in the 27,3% of cases, teeth with no periodontal disease in the 23,6% of cases, teeth under supportive periodontal treatment in the 9,1% of cases, a fixed full-arch prosthesis supported by more than 4 implants in the 3,6% of cases, and an overdenture in the 1,8% of cases.

Considering maxillary restorations, the opposing arch was in the 51,7% of cases an All-on-four prosthesis, in the 31,0% of cases teeth with no periodontal disease, and in the 17,3% of cases teeth under supportive periodontal treatment.

The overall follow-up range was from 12 to 130 months after surgery (mean 63,2 months).

The follow-up range for the mandibular restorations was from 12 to 130 months after surgery (mean 66,7 months) while the follow-up range for the maxillary restorations was from 12 to 100 months after surgery (mean 56,3 months).

The patient-related prevalence of peri-implant mucositis and peri-implantitis is shown in Table 1 for mandibular restoration and in Table 2 for the maxillary ones.

**Figure 1:**
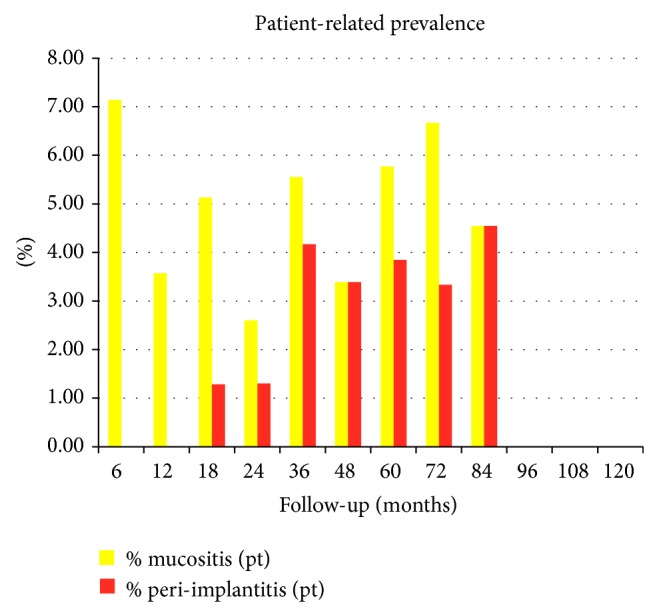
Overall patient-related prevalence of peri-implant mucositis and peri-implantitis.

The overall percentage of patient-related prevalence of peri-implant mucositis and peri-implantitis is shown in Figure 1. The prevalence of peri-implant mucositis varied from the 7,14% of patients at 6 months to 0% from 96 to 120 months after surgery, while the prevalence of peri-implantitis ranged from the 4,55% of patients at 84 months to the 0% at 6, 12, 96, 108, and 120 months after surgery.

**Figure 2:**
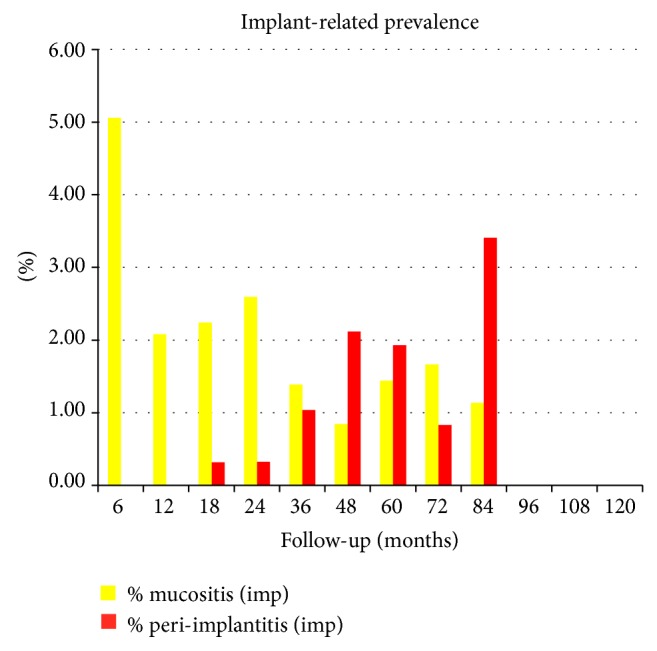
Overall implant-related prevalence of peri-implant mucositis and peri-implantitis.

The implant-related prevalence of peri-implant mucositis, peri-implantitis, and implant loss is shown in Table 3 for mandibular restoration and in Table 4 for the maxillary ones. The overall percentage of implant-related prevalence of peri-implant mucositis and peri-implantitis is shown in Figure 2. It ranged from the 5,06% after 6 months to the 0% after 96, 108, and 120 months from surgery for peri-implant mucositis and from the 3,81% after 84 months to the 0% after 6, 12, 96, 108, and 120 months from surgery for peri-implantitis.

No significant differences were found between axial and tilted implants.

**Figure 3:**
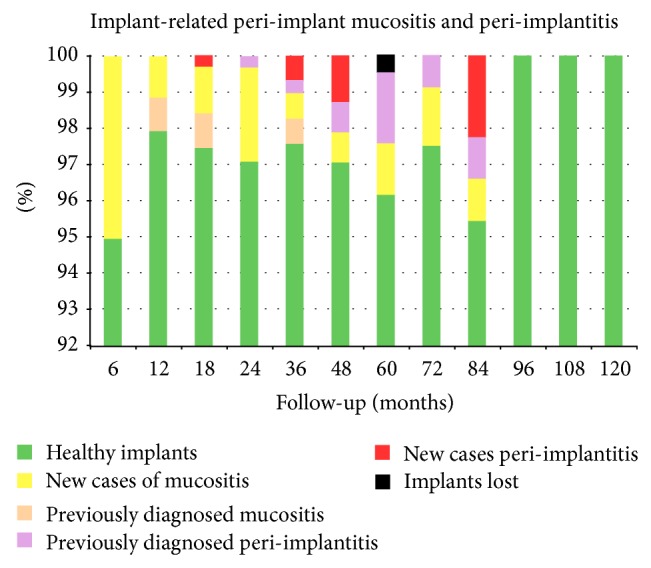
Implant related prevalence and incidence of peri-implant mucositis, peri-implantitis, and implants' loss.

The overall implant-related incidence of peri-implant mucositis, peri-implantitis, and implant loss is shown in Table 5 and Figure 3.

One implant was lost and recorded at the 60th month of follow-up. The incidence of peri-implant mucositis was highest at the 6th month of follow-up with the 5,06% of implants, while at the 96th, 108th, and 120th month of follow-up it affected the 0% of them. The incidence of peri-implantitis ranged from the 2,27% of implants after 84 months to the 0% of implants after 6, 12, 24, 60, 72, 96, 108, and 120 months of surgery.

The median follow-up time for the incidence of peri-implant mucositis was 18 months and for peri-implantitis was 48 months.

Patient-related and implant-related cumulative rates of having experienced at least 1 episode of peri-implant mucositis or peri-implantitis are shown in Tables 6 and 7.

No significant differences were found between smoker and no-smoker patients.

Episodes of peri-implantitis were documented in 3 patients.

All 3 patients had an All-on-Four restoration supported by Nobel-Speedy Groovy implants.

Patient 1 was a 54-year-old no-smoker woman. She presented prior history of periodontitis and was under supportive periodontal therapy on the teeth of the opposing jaw. She was found with one side of a mesial implant with PD = 5 mm and a radiographic mild bone loss in the 36 months' follow-up visit. Episodes of peri-implant mucositis were not recorded before.

**Figure 4:**
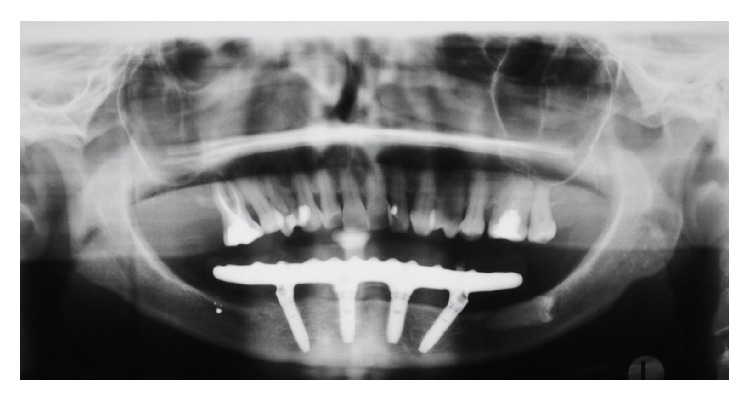
Orthopantomography of the patient 6 months after surgery.

**Figure 5:**
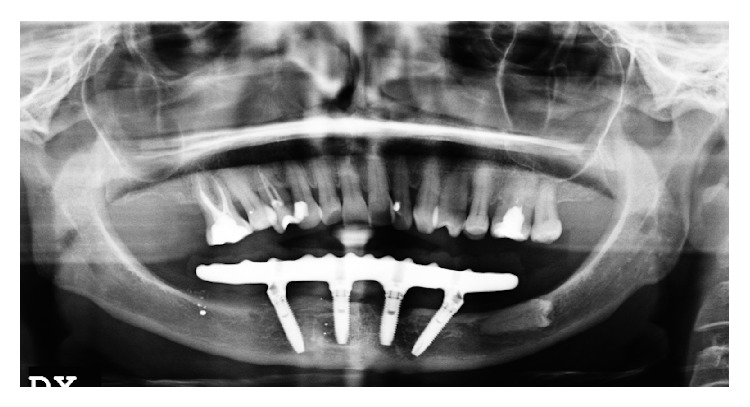
Orthopantomography of the patient after 36 months from surgery.

**Figure 6:**
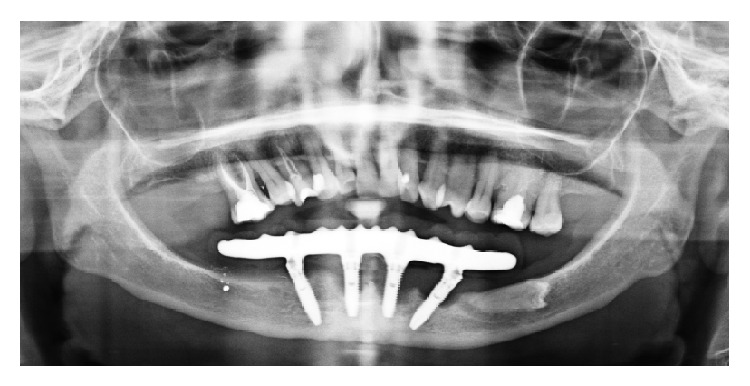
Orthopantomography of the patient after 84 months from surgery.

**Figure 7:**
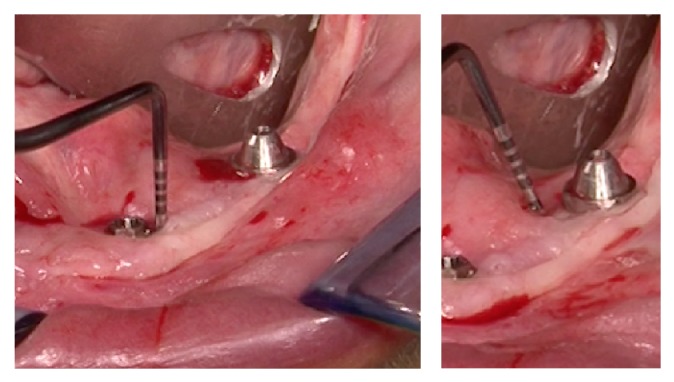
Clinical probing of the peri-implant pockets.

**Figure 8:**
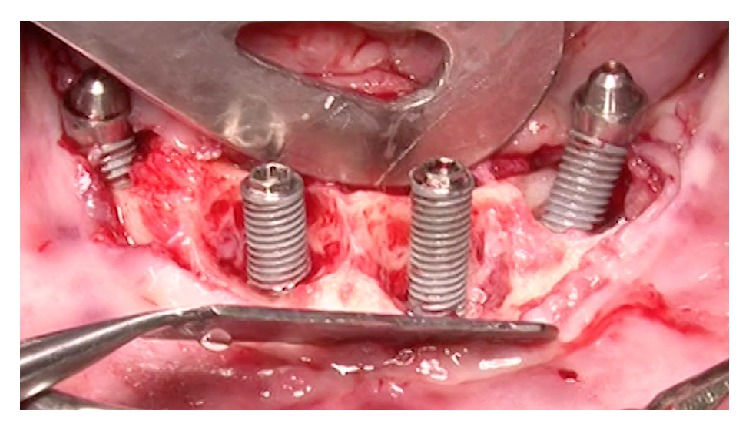
Surgical access to the peri-implant bone defects.

Following a maintenance protocol the situation remained unchanged until the 84 months' follow-up visit in which the peri-implant pocket deepened and peri-implantitis was diagnosed also in other 2 implants (Figures 4–8).

Patient 2 was a heavy smoking 62-year-old male. He had prior history of periodontitis and on the opposing jaw he had a complete removable denture. At the 18 months' follow-up visit he was found with 3 sides of a mesial implant with PD of 8, 7, and 5 mm, a radiographic bone loss, and peri-implant mucositis in the adjacent implant. Episodes of peri-implant mucositis were not recorded before. At the 24 months' follow-up visit the PD of the implant affected by peri-implantitis had still 2 sides with PD of 7 mm and 5 mm and after 36 months only one side with PD of 5 mm while the other implants were healthy. At the 48 months' follow-up all implants were diagnosed with peri-implantitis and at the 58th month one mesial implant was lost due to peri-implantitis.

Patient 3 was a 54-year-old heavy smoking woman. She had previous history of periodontitis and was under supportive periodontal therapy on the teeth of opposing arch. At the 36 months' follow-up visit she was found with all sides of a tilted implant with PD between 5 mm and 9 mm a radiographic bone loss. Episodes of peri-implant mucositis were not recorded before.

## 4. Discussion

Many experiments in animals and studies in humans showed that plaque formation at implant level resulted in peri-implant mucositis [24–26].

In the present study the patient-related prevalence of mucositis was lower than the 8% in any follow-up visit and it was higher in the first follow-up visit (6 months) than in the following ones. These findings are in agreement with Östman et al. [27] but in disagreement with several studies that observed a frequency remarkably higher of peri-implant mucositis around the 80% of patients [19, 28–33]. These studies examined a great variability of rehabilitations and implants and reported the bleeding of probing alone as presence of the pathology. However the bleeding on probing on its own could overestimate the presence of gingival inflammation in periodontally healthy subjects [34]; it could be strongly influenced by the operator and could lead to a high rate of false positive values [19]. For this reason in the present study an implant was considered affected by peri-implant mucositis if the bleeding score was >1 as indicated in other studies [23].

The fact that the prosthesis is screw-retained nullifies the risk of infection due to submucosal persistence of luting cement [35].

Mombelli et al. [19] based on the reviewed articles stated that the prevalence of peri-implantitis seemed to be around the 10% of implants and the 20% of patients during 5–10 years.

However this statement had to be taken with caution because of the lack of uniformity in the definition, thresholds for peri-implantitis, and differences in the composition of the population among the epidemiological and risk factor studies as pointed out by several reviews [18–20, 29].

In the present study however the prevalence of peri-implantitis was lower than 4,5% relating to patients and lower than 3,5% related to implants.

Moreover all patients that developed peri-implantitis were not diagnosed with peri-implant mucositis in the previous follow-up visit. This could suggest that a visit every 6 months in the first 2 years and yearly after may be not enough frequent to prevent it properly.

By choosing such a generous definition of peri-implantitis in the first 24 months it could be possible to see some alterations that include bone loss related to re-establishment of biologic width. It seems more feasible that signs of peri-implantitis might not really begin to show until the 36 months' follow-up or later.

Peri-implantitis did not regress in any patient or implant. The absence of peri-implantitis in the latest follow-up visits is due to the limited number of patients that reached the 96, 108, and 120 months' of follow-up by now.

This study is not in accordance with Marrone et al. [36] who in a survey over 133 patients found a strong association between peri-implant disease and total edentulism, but the population considered in that survey was very small, only 7 totally edentulous subjects.

In the present study all patients that developed peri-implantitis had a prior history of periodontitis. This is in accordance with many previous studies [18, 19, 29, 37, 38] even if the sample size was limited and the groups of patients with and without history of periodontitis were not homogeneous.

Most of the studies concerning risk factors of peri-implant disease concluded that smoking was distinctly involved [39–42]. However the present study did not find such an association even though 2 of the 3 patients that presented peri-implantitis were heavy smokers. This discrepancy from literature could be explained either by the small sample size or by smoking that could have masked symptoms of peri-implant mucositis reducing vascularization of soft tissues and the bleeding on probing.

## 5. Conclusions

The use of immediate loaded full-arch prosthesis supported by two mesial axial implants and two distal tilted implants is a viable rehabilitation option, considering the lower rate of peri-implant mucositis and peri-implantitis compared to what was found in literature.

However a meticulous attention to the hygienic conditions and the adoption of a systematic follow-up schedule are necessary.

Further long-term studies are needed in order to achieve a better understanding of risk factors for peri-implant mucositis and peri-implantitis and validate effective preventive and therapeutic protocols.

## Figures and Tables

**Table 1:** Patient-related prevalence in the mandible.

Follow-up	*N*° patients	*N*° mucositis (pt)	*N*° peri-implantitis (pt)
6	55	5 (9,09%)	0
12	55	3 (5,45%)	0
18	50	4 (8%)	1 (2%)
24	50	1 (2%)	1 (2%)
36	47	2 (4,26%)	2 (4,26%)
48	42	2 (4,76%)	2 (4,76%)
60	37	3 (8,11%)	2 (5,41%)
72	22	2 (9,09%)	1 (4,55%)
84	18	0	1 (5,56%)
96	7	0	0
108	6	0	0
120	2	0	0

**Table 2:** Patient-related prevalence in the maxilla.

Follow-up	*N*° patients	*N*° mucositis (pt)	*N*° peri-implantitis (pt)
6	29	1 (3,45%)	0
12	29	0	0
18	28	0	0
24	27	1 (3,70%)	0
36	25	2 (8,00%)	1 (4,00%)
48	17	0	0
60	15	0	0
72	8	0	0
84	4	1 (25,00%)	0
96	2	0	0

**Table 3:** Implant-related prevalence in the mandible.

Follow-up	*N*° implants	*N*° mucositis (imp)	*N*° peri-implantitis (imp)	Impl. lost
6	220	13 (5,91%)	0	0
12	220	7 (3,18%)	0	0
18	200	7 (3,50%)	1 (0,50%)	0
24	200	4 (2,00%)	1 (0,50%)	0
36	188	2 (1,06%)	2 (1,06%)	0
48	168	2 (1,19%)	5 (2,98%)	0
60	147	3 (2,04%)	4 (2,72%)	1 (0,68%)
72	88	2 (2,27%)	1 (1,14%)	0
84	72	0	3 (4,17%)	0
96	28	0	0	0
108	24	0	0	0
120	8	0	0	0

**Table 4:** Implant-related prevalence in the maxilla.

Follow-up	*N* implants	*N*° mucositis (imp)	*N*° peri-implantitis (imp)
6	116	4 (3,45%)	0
12	116	0	0
18	112	0	0
24	108	4 (3,70%)	0
36	100	2 (2,00%)	1 (1,00%)
48	68	0	0
60	60	0	0
72	32	0	0
84	16	1 (6,25%)	0
96	8	0	0

**Table 5:** Incidence of peri-implant mucositis and peri-implantitis.

Follow-up	Total implants	Incidence mucositis	Incidence peri-implantitis	Imp. lost
6	336	17 (5,06%)	0	0
12	336	4 (1,19%)	0	0
18	312	4 (1,28%)	1 (0,32%)	0
24	308	8 (2,60%)	0	0
36	288	2 (0,69%)	2 (0,69%)	0
48	236	2 (0,85%)	3 (1,27%)	0
60	207	3 (1,45%)	0	1 (0,48%)
72	120	2 (1,67%)	0	0
84	88	1 (1,14%)	2 (2,27%)	0
96	36	0	0	0
108	24	0	0	0
120	8	0	0	0

**Table 6:** Patient-related experience of peri-implant mucositis and peri-implantitis rate.

Follow-up	*N* patients	Experience of mucositis	Experience of peri-implantitis
6	84	6 (7,14%)	0
12	84	8 (9,52%)	0
18	78	10 (12,82%)	1 (1,28%)
24	77	11 (14,29%)	1 (1,30%)
36	72	13 (18,06%)	3 (4,17%)
48	59	15 (25,42%)	2 (3,39%)
60	52	16 (30,77%)	2 (3,85%)
72	30	11 (36,67%)	1 (3,33%)
84	22	10 (45,45%)	1 (4,55%)
96	9	5 (55,56%)	0
108	6	3 (50,00%)	0
120	2	1 (50,00%)	0

**Table 7:** Implant-related experience of peri-implant mucositis and peri-implantitis rate.

Follow-up	*N* implants	Experience of mucositis	Experience of peri-implantitis
6	336	17 (5,06%)	0
12	336	21 (6,25%)	0
18	312	21 (6,73%)	1 (0,32%)
24	308	28 (9,09%)	1 (0,32%)
36	288	30 (10,42%)	3 (1,04%)
48	236	32 (13,56%)	5 (2,12%)
60	207	30 (14,49%)	5 (2,42%)
72	120	16 (13,33%)	1 (0,83%)
84	88	13 (14,77%)	3 (3,41%)
96	36	5 (13,89%)	0
108	24	3 (12,50%)	0
120	8	1 (12,50%)	0
